# Sex- and age-associated factors drive the pathophysiology of MASLD

**DOI:** 10.1097/HC9.0000000000000523

**Published:** 2024-08-26

**Authors:** Ajay K. Yadav, Justin J. MacNeill, Aleksei Krylov, Nadia Ashrafi, Romana Ashrafi Mimi, Romil Saxena, Sheng Liu, Stewart F. Graham, Jun Wan, Núria Morral

**Affiliations:** 1 Department of Medical and Molecular Genetics, Indiana University School of Medicine, Indianapolis, Indiana, USA; 2 Metabolomics Department, Corewell Health Research Institute, Royal Oak, Michigan, USA; 3 Corewell Health William Beaumont University Hospital, Royal Oak, Michigan, USA; 4 Department of Pathology and Laboratory Medicine, Emory University School of Medicine, Atlanta, Georgia, USA; 5 Oakland University-William Beaumont School of Medicine, Rochester, Michigan USA; 6 Center for Computational Biology and Bioinformatics, Indiana University School of Medicine, Indianapolis, Indiana, USA; 7 Department of Biochemistry and Molecular Biology, Indiana University School of Medicine, Indianapolis, Indiana, USA

## Abstract

**Background:**

Metabolic dysfunction–associated steatotic liver disease (MASLD) is strongly associated with obesity. Sex and age affect MASLD prevalence and pathophysiology. The use of animal models fed Western-style diets is vital for investigating the molecular mechanisms contributing to metabolic dysregulation and for facilitating novel drug target identification. However, the sex-associated and age-associated mechanisms underlying the pathophysiology remain poorly understood. This knowledge gap limits the development of personalized sex-specific and age-specific drug treatments.

**Methods:**

Young (7 wk) and aged (52 wk) male and female mice were fed a high-fat diet (HFD) or low-fat diet. Liver metabolome (>600 molecules) and transcriptome profiles were analyzed.

**Results:**

Male and female mice fed an HFD developed obesity, glucose intolerance, and hepatic steatosis. However, fasting blood glucose, insulin, and serum alanine aminotransferase levels were higher in males fed an HFD, indicating a more severe metabolic disease. In addition, males showed significant increases in liver diacylglycerides and glycosylceramides (known mediators of insulin resistance and fibrosis), and more changes in the transcriptome: extracellular matrix organization and proinflammatory genes were elevated only in males. In contrast, no major increase in damaging lipid classes was observed in females fed an HFD. However, aging affected the liver to a greater extent in females. Acylcarnitine levels were significantly reduced, suggestive of changes in fatty acid oxidation, and broad changes in the transcriptome were observed, including reduced oxidative stress response gene expression and alterations in lipid partitioning genes.

**Conclusions:**

Here, we show distinct responses to an HFD between males and females. Our study underscores the need for using both sexes in drug target identification studies, and characterizing the molecular mechanisms contributing to the MASLD pathophysiology in aging animals.

## Graphical abstract

**Figure:**
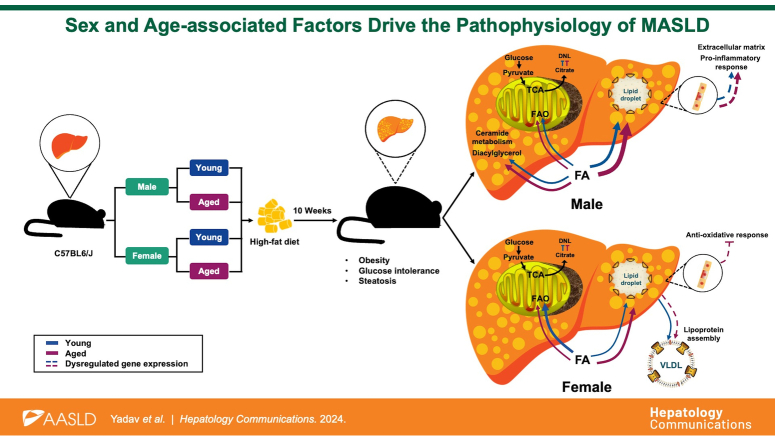

## INTRODUCTION

Metabolic dysfunction–associated steatotic liver disease (MASLD) is a risk factor for type 2 diabetes and cardiovascular disease.^1–3^ Aging of the global population together with high obesity rates, promoted by sedentarism and overnutrition, are the major contributors to its growing prevalence, currently estimated to be 30% in adults.^4^ In the early stages, MASLD is characterized by the presence of steatosis without evidence of hepatocellular damage (metabolic dysfunction–associated steatotic liver, MASL). MASL is strongly linked to insulin resistance.^5^ Approximately 20% of patients with simple steatosis progress to metabolic dysfunction–associated steatohepatitis, a condition characterized by steatosis, inflammation with hepatocyte injury, and fibrosis, which can progress to cirrhosis, cancer, and liver failure.^6^

The liver plays a central role in multiple aspects of physiology, including innate immunity, detoxification, bile synthesis, protein synthesis, and the regulation of carbohydrate and lipid metabolism. In response to external stimuli, the liver activates or suppresses entire gene networks to perform its diverse functions.^7^ Aberrant transcriptional control of gene expression constitutes a central aspect of the pathophysiology of MASLD. Excessive calorie consumption from carbohydrates and fats are overriding factors of energy imbalance, signaling changes in gene expression. Sex is an important determinant of gene expression and responses to external signals.^8^ In addition, aging is associated with the loss of metabolic elasticity due to a decline in the ability of the liver to adjust its gene expression programs,^9^ adding another factor that drives the pathophysiology. Epidemiology studies have reported that men experience a higher prevalence of MASLD than premenopausal women.^10,11^ However, postmenopausal women have a similar incidence of MASLD relative to men and display a faster progression of the disease, placing them at a similar or even higher risk for the development of cardiovascular disease.^10,11^

The use of animal models is vital for investigating the molecular mechanisms leading to metabolic dysfunction and facilitating the development of new drug treatments. Nevertheless, most studies identifying the molecular mechanisms underlying MASLD pathophysiology have been conducted in young male mice. Thus, the response of the liver to Western-style diets in females and aged animals remains poorly characterized. In this study, we aimed to identify sex-related and age-related differences in the metabolome and transcriptome that drive the etiology of MASLD. Our data indicate that the accrual of lipid classes known to induce cellular toxicity, together with the activation of proinflammatory responses, are the main drivers of the pathophysiology in males, while in females, changes in the transcriptome associated with aging are the major determinants.

## METHODS

### Animals

All animal procedures were conducted in accordance with the National Institutes of Health guidelines and were approved by the Indiana University School of Medicine Institutional Animal Care and Use Committee. C57BL6/J male and female, 7- and 52-week-old mice were purchased from The Jackson Laboratory. A standard 12-hour light/12-hour dark cycle (7 am/7 pm) was maintained throughout the experiments. Mice were fed (i) a high-fat diet (HFD) (D12492, Research Diets: 60 kcal% fat, 20% protein, 20% carbohydrate; contains 7% kcal from sucrose, as well as 279.6 mg/kg cholesterol from lard; 5.24 kcal/g), which promotes hepatic steatosis in male C57BL/6J mice,^12^ or (ii) a matched control diet (D12450K: 10 kcal% fat, 20% protein, 70% carbohydrate; no sucrose, and 51.6 mg/kg of cholesterol; 3.85 kcal/g), for 10 weeks (n = 8/group). Mice were kept in a BSL1 room and had free access to food and water. Fresh pellets were provided weekly, and body weights were monitored. The average food consumption of C57BL/6 mice was estimated by measuring leftover food from the previous week (starting on week 2, 8 total measurements) divided by the number of mice in the cage. Blood glucose measurements under fed conditions were taken from 9:00 to 9:45 am. Mice were euthanized by decapitation under fed (ad libitum) conditions. Tissues were collected and snap-frozen in liquid nitrogen, embedded in optimal cutting temperature compound, and frozen in liquid nitrogen, or fixed in 10% buffered formalin for histological analysis.

### RNA-seq

Total RNA was extracted using an RNeasy Midi kit (Qiagen) following the manufacturer’s protocol (n = 4/group). RNA integrity was evaluated by RNA ScreenTape analysis (Agilent Technologies). RNA library construction and sequencing were performed by the Center for Medical Genomics at Indiana University School of Medicine. Briefly, mRNA libraries were generated from 100 ng RNA using the KAPA mRNA Hyperprep kit (Roche). Paired-end 100-bp reads were generated using the Illumina NovaSeq 6000 platform (~45 million reads/sample). Nearly 85% of reads were uniquely mapped to the mouse genome reference mm10 using STAR (Spliced Transcripts Alignment to a Reference).^13^ To evaluate the quality of the RNA-seq data, the number of reads that fell into different annotated regions (exonic, intronic, splicing junction, intergenic, promoter, UTR, etc.) of the reference genes was determined using bamUtils.^14^ Low-quality mapped reads (including reads mapped to multiple positions) were excluded, and featureCounts^15^ was used to quantify gene expression. The data were filtered using a read count >10 in at least 2 of the samples, normalized using the TMM (trimmed mean of M values) method, and subjected to differential expression analysis using edgeR.^16^ Differentially expressed genes (false discovery rate <0.05) between the groups were analyzed separately for males and females. Ingenuity pathway analysis^17^ was used for comparisons between young and aged mice.

### Metabolomics analysis

#### Liver sample preparation using MxP Q500

Liver tissue samples were prepared as per the manufacturer’s instructions (Biocrates Life Sciences, AG). Liver tissue samples and calibration standards were thawed on ice. Liver raw tissue samples were extracted using 100% isopropanol. Samples were subsequently homogenized for 30 seconds for 3 times between 60-second intervals and centrifuged at 15,000*g* at 4°C for 10 minutes to collect the extracted supernatant. Calibration standards and quality controls were dissolved in 100 μL of H_2_O and mixed at 1200 rpm for 15 minutes. Twenty microliters of liver samples, 10 μl of calibration standards, quality controls, and phosphate buffer solution were added to the 96-well plate. The plate was dried under nitrogen for 30 minutes. All samples and standards were included in a premix of phenylisothiocyanate at room temperature for 60 minutes for derivatization purposes and subsequently dried under nitrogen for 60 minutes. Samples were extracted in 5 mM ammonium acetate in methanol for 30 minutes using an orbital shaker, and the extracts were collected by centrifuging the preparation plate at 500*g* for 2 minutes. Sample extracts were diluted with H_2_O (1:1) for the liquid chromatography phase of the analysis. For the flow injection analysis (FIA), in a separate plate, 70 μL of sample extract was mixed with 430 μL of the kit solvent, and 10 μL of quality control sample extract was mixed with 490 μL of the kit solvent. Liquid chromatography and FIA plates were sealed, mixed for 10 minutes at 600 rpm at room temperature, and placed into the thermostatically controlled autosampler for analysis.

#### DI-ultra performance liquid chromatography-MS/MS

Sample extracts were analyzed using a Waters I-class ultra performance liquid chromatography unit coupled with a Waters Xevo-TQ-S (Waters Corporation). For ultra performance liquid chromatography analysis, sample extracts were separated using the MxP Quant 500 C18 column with an attached guard and precolumn mixer (Biocrates Life Sciences). The mobile phase consisted of A: H_2_O and formic acid (0.2%); B: MeCN and formic acid (0.2%) delivered at a flow rate of 0.8 mL/min with a gradient of B: 0–100% over 4.50 minutes. Eluent %B was increased to 1.00 mL/min flow rate and maintained at 100% for 30 seconds, followed by a rapid return to the initial conditions for 70 seconds to equilibrate the column. Both positive and negative mode gradients were 5.80 minutes long. The negative mode acquisition gradient differed from the positive mode with a difference in %B composition between 2.00 and 4.50 minutes. The injection volume was 5 μL for positive data acquisition and 15 μL for the negative run. The wash solvent composition consisted of H_2_O: MeOH: MeCN: isopropanol (vol/vol).

#### FIA-MS/MS analysis

Q500 Kit offers direct flow injections (FIA) for lipid analysis. An isocratic method was performed using the kit-provided solvent (290 mL MeOH: 1 ampule of FIA additives). The isocratic mobile phase (B: 100% MeOH) was delivered at a low flow rate of 0.03 mL/min. The injection volume was 20 μL for both positive and negative mode acquisitions. All data were extracted using the MetIDQ software following Biocrates’ instructions (Biocrates).

#### Data analysis

The MetaboAnalyst (v5.0) platform was used to generate heat maps and volcano plots of the normalized data.^18^

### Statistical analysis

GraphPad Prism v10.1.1 was used to generate graphs and to calculate *p* values by one-way ANOVA with Šídák’s correction for multiple comparisons. A *p* value of <0.05 was considered statistically significant. Data are presented as the arithmetic mean ± SD or SEM.

See Supplemental Methods, http://links.lww.com/HC9/B20, for details on serum biochemistries, tissue histology, and western blot analysis.

## RESULTS

### High-fat diet feeding induces a worse metabolic outcome in males than females and is aggravated by age

**FIGURE 1:**
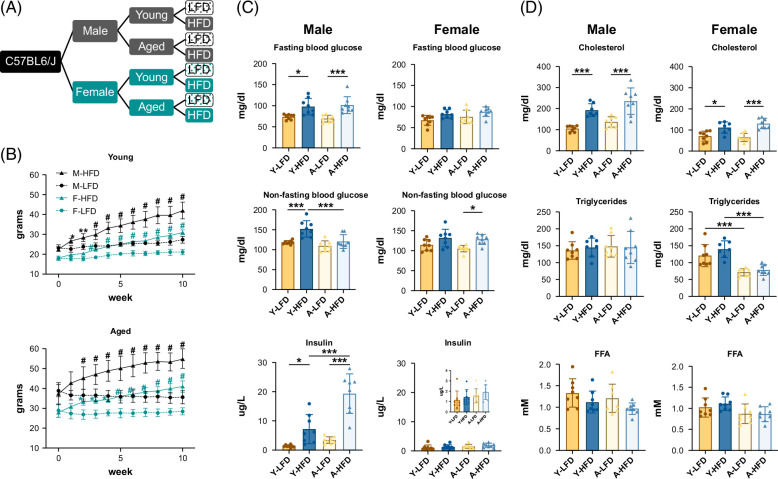
Experimental design, body weight, and serum chemistries. (A) Experimental design (n = 8). (B) Body weight gain over the course of 10 weeks. Bars represent SD. *p < 0.05, **p < 0.01, ^#^ *p* < 0.001. (C) Blood glucose and serum insulin (n = 8). (D) Serum triglycerides, FFA, and cholesterol (n = 7–8). Bars represent SD. *p < 0.05, **p < 0.01, ***p < 0.001. Abbreviation: FFA, free fatty acids.

To investigate the influence of sex and age on the pathophysiology of MASL, male and female, young (7 wk) and aged (52 wk), C57BL/6 mice were fed an HFD or a low-fat control diet for 10 weeks (Figure 1A). This animal model displays the metabolic dysfunction associated with MASL.^12,19,20^ Aged female mice have a ~3-fold reduction in estradiol relative to young female mice^21^ and are a relevant model to study the effects of reduced estrogen and cellular aging. As anticipated, mice fed an HFD gained significantly more weight than mice fed the control diet in both sexes and age groups (Figure 1B, Supplemental Figure S1A, http://links.lww.com/HC9/B21). In young males and females, no differences in food consumption were observed between mice fed the HFD and those fed the low-fat diet (LFD). Nevertheless, aged mice (male and female) on the HFD consumed slightly more food than young mice (Supplemental Figure S1B, http://links.lww.com/HC9/B21).

In males, fasting glucose in the HFD group was significantly higher than in the LFD group, indicative of glucose intolerance (Figure 1C), whereas in females, there was only a trend. In addition, HFD-fed male mice showed marked hyperinsulinemia, which was more prominent in aged animals (Figure 1C). Non-fasting glucose levels were also significantly higher in the HFD-fed young male group. Remarkably, aged male mice fed an HFD showed a trend toward higher non-fasting glucose levels, likely due to the high insulin levels in this group (Figure 1C). In female mice (young and aged), insulin levels were not significantly elevated by the HFD. In contrast to aged male mice, non-fasting blood glucose levels were significantly elevated in aged females fed an HFD (Figure 1C). Glucose tolerance tests revealed impaired glucose tolerance in young and aged male mice (Supplemental Figure S1C, http://links.lww.com/HC9/B21). In females, impaired glucose tolerance was observed to a greater extent in young mice than in aged mice (Supplemental Figure S1C, http://links.lww.com/HC9/B21). Altogether, these data suggest that females develop glucose intolerance when fed an HFD, albeit with less severity than males.

Serum cholesterol levels were significantly increased in both sexes, reflecting the higher cholesterol content of the HFD (Figure 1D). Serum triglyceride levels were not different between groups in males and were lower in aged mice than in young female mice. No changes in free fatty acids were observed in either the male or female mice (Figure 1D).

### Distinct histology in males and females

**FIGURE 2:**
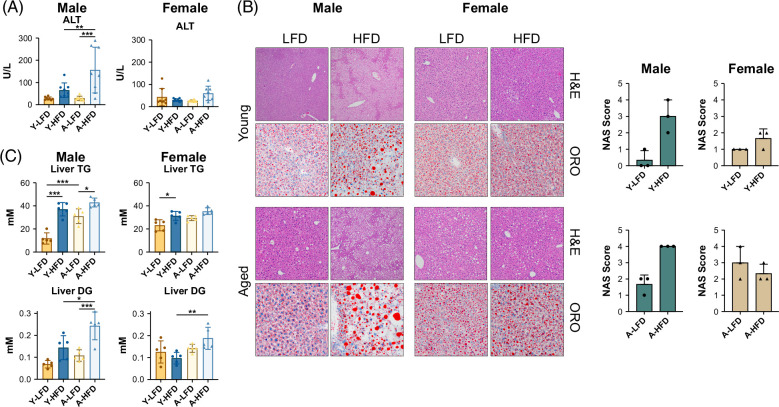
Serum ALT and liver histology. (A) Serum ALT levels (n = 8). (B) Hematoxylin/eosin and Oil Red O staining of liver sections. NAS scores (sum of steatosis, hepatocellular ballooning, and lobular inflammation scores) are shown on the right (n = 3). Hematoxylin/eosin, ×100; Oil Red O, ×200. (C) Liver triglycerides and diacylglycerides were quantified in a targeted metabolomics assay (n = 5). Bars represent SD. *p < 0.05, **p < 0.01, ***p < 0.001. Abbreviations: ALT, alanine aminotransferase; NAS, nonalcoholic fatty liver disease activity score.

Consistent with a more severe metabolic disease in males, the liver function marker ALT (alanine aminotransferase) was elevated in mice fed an HFD, particularly in aged animals (Figure 2A), suggesting liver damage. In liver sections stained with hematoxylin/eosin, 3 types of changes were observed: (1) slight clearing of the cytoplasm of hepatocytes, which corresponded to very small lipid granules on Oil Red O staining; (2) enlargement of hepatocytes by numerous small vacuoles of fat (microvacuolar steatosis); and (3) presence of 1–4 medium or large vacuoles of fat within variably enlarged hepatocytes (macrovacuolar steatosis). The patterns of fat accumulation appeared to be distinct between males and females. Young males and females on the LFD showed the first pattern on hematoxylin/eosin staining, which represents the baseline in mice in the fed state, and an NAFLD activity score (NAS)^22^ of ≤1 (Figure 2B). On an HFD, young male mice showed marked microvacuolar steatosis involving 60%–80% of hepatocytes and macrovacuolar steatosis involving 10% of hepatocytes (NAS score of 2–4), while young female mice on an HFD showed a similar pattern of steatosis to LFD-fed mice, with only mild changes (NAS score ≤2). On an HFD, older males showed microvacuolar (70% hepatocytes) and macrovacuolar steatosis (10%–20% hepatocytes, large droplet size) and a NAS score of 4. Older females on an LFD showed macrovacuolar steatosis (20%–60%, medium droplet size), and feeding an HFD changed this pattern only mildly (NAS score of ~3). No fibrosis was observed in males or females, as anticipated in this mouse model.^12,20^ A few aged female mice showed large foci of monotonous lymphoid cells, suggesting lymphoproliferative disease/lymphoma, independent of diet.

### Sex dimorphism in the liver metabolome

To assess the impact of diet, sex, and age on liver metabolism, profiles of more than 600 molecules were obtained, including 523 lipids and 107 small molecules. Notable differences were observed between the groups, in particular between males and females. Triglycerides increased in male mice and young females fed HFD (Figure 2C). However, young male mice showed an increase in all triglyceride subclasses, while females showed an increase in several triglyceride subclasses and a decrease in others (Supplemental Figures S2A, B, http://links.lww.com/HC9/B21). Remarkably, diacylglycerol, a molecule implicated in insulin resistance in the liver,^5^ was significantly increased in aged male mice fed an HFD relative to the LFD. In females, the changes between the LFD and HFD were not significant (Figure 2C and Supplemental Figure S2C, http://links.lww.com/HC9/B21).

**FIGURE 3:**
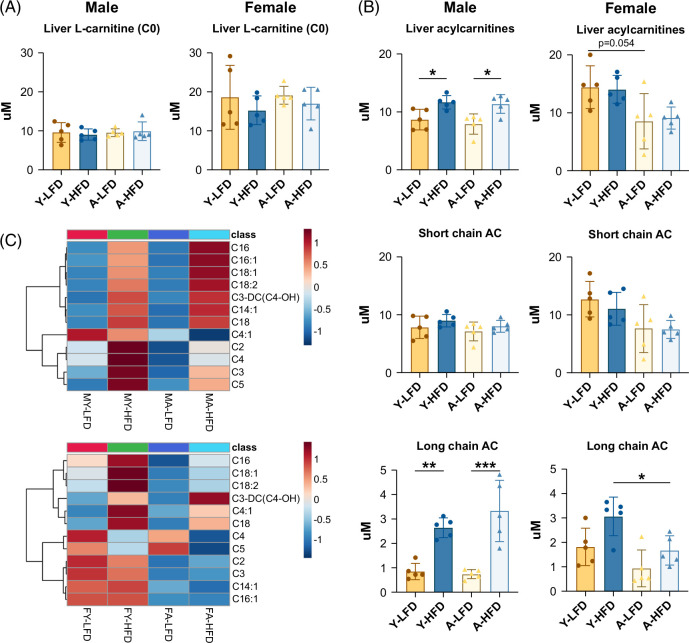
Liver acylcarnitines. (A) Levels of l-carnitine (C0). (B) Total acylcarnitines; short-chain and long-chain acylcarnitines. (C) Heatmap showing changes in acylcarnitines in males and females fed the LFD or HFD. Bars represent SD. *p < 0.05, **p < 0.01, ***p < 0.001. Abbreviations: HFD, high-fat diet; LFD, low-fat diet.

Acylcarnitines are generated from the conjugation of fatty acids with l-carnitine, and their main function is the transport of acyl groups through the mitochondrial membrane for oxidation in the matrix. l-carnitine (C0) was higher in females than in males and was not affected by diet or age (Figure 3A). Notably, feeding an HFD increased the overall levels of acylcarnitines to a similar degree in young and aged males, mostly due to an increase in long-chain fatty acid subclasses. No changes in acylcarnitines were observed in aged mice compared to young male mice (Figures 3B, C). In contrast, the overall levels of acylcarnitines in females were not affected by the HFD (Figures 3B, C). However, in aged females, there was a clear trend for lower levels of acylcarnitines (*p* = 0.054; Figure 3B). Furthermore, significantly lower levels of long-chain acylcarnitines were observed in aged relative to young females fed the HFD (Figure 3B).

**FIGURE 4:**
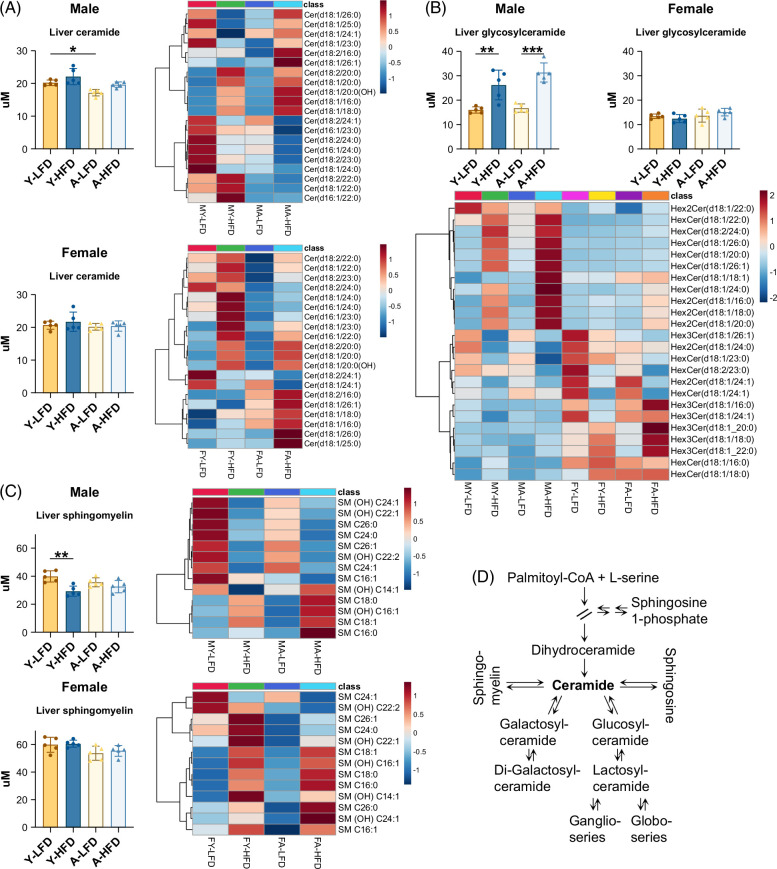
Liver sphingolipids. (A) Liver ceramides and heatmap of ceramide subclasses. (B) Liver glycosylceramides and heatmap of the classes detected. (C) Liver sphingomyelin. (D) Schematic of sphingolipid synthesis.^23^ Bars represent SD. *p < 0.05, **p < 0.01, ***p < 0.001.

Sphingolipid profiles were also remarkably different between the sexes. Overall, the levels of ceramides were not affected by the HFD. Nevertheless, the abundance of specific subclasses shifted with diet and age in both sexes (Figure 4A, Supplemental Figure S3A, http://links.lww.com/HC9/B21), changing the ratio between very-long-chain and long-chain ceramides (Supplemental Figure S3A, http://links.lww.com/HC9/B21). In males fed an HFD, this ratio changed to a higher extent than in females. Two of the long-chain ceramides that increased, C16:0 and C18:0 (Supplemental Figure S3A, http://links.lww.com/HC9/B21), have been associated with insulin resistance.^24,25^ Furthermore, glycosylceramides were robustly increased in young and aged male mice fed an HFD, whereas no changes were observed in females (Figure 4B). The increase included glucosylceramides (HexCer) and di-galactosylceramide (Hex2Cer) (Figure 4B, Supplemental Figure S3B, http://links.lww.com/HC9/B21). Dihydroceramide, a metabolite upstream of the ceramide synthesis pathway (Figure 4D), did not increase in males upon HFD feeding and decreased in aged female mice (Supplemental Figure S3C, http://links.lww.com/HC9/B21). Sphingomyelin, a major component of the plasma membrane^26^ (Figure 4D), was more abundant in females than in males. Young male mice fed an HFD displayed reduced levels, but no effects were observed in aged males or females (Figure 4C).

**FIGURE 5:**
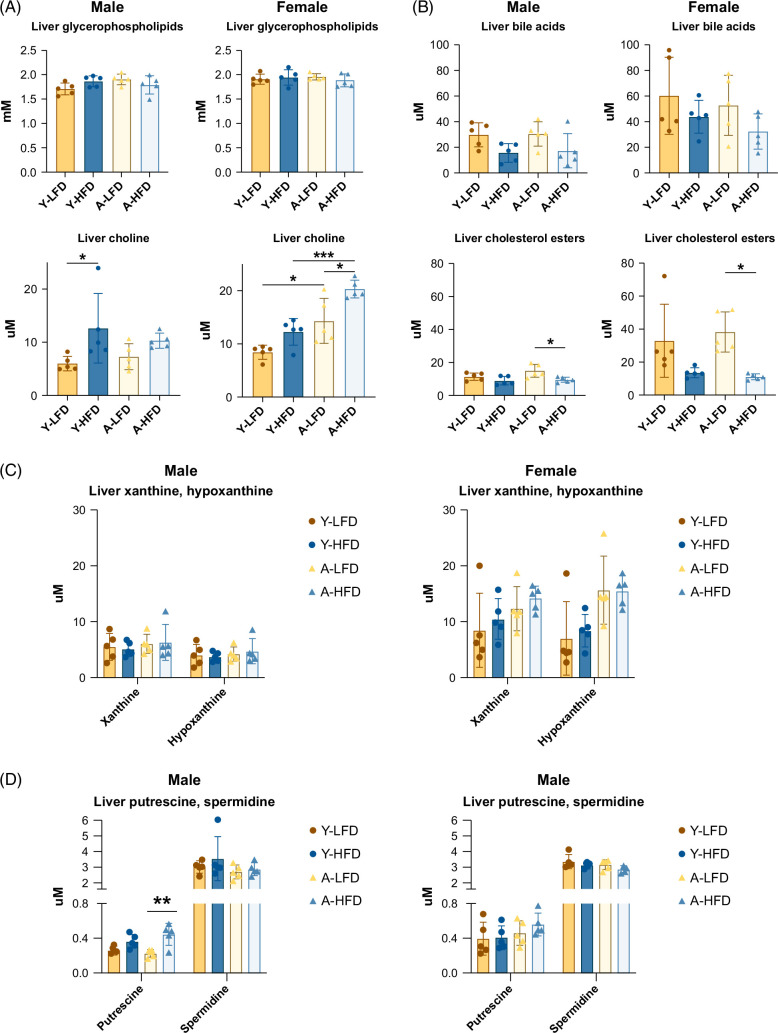
Liver glycerophospholipids, bile acids, cholesterol esters, nucleobase-related molecules, and biogenic amines. (A) Glycerophospholipids (phosphatidylcholine plus lysophosphatidylcholine) and choline levels in liver tissue. (B) Overall levels of bile acids (TCA, TDCA, TCDCA, and TMCA) and liver cholesterol esters. (C) Xanthine and hypoxanthine, products of the catabolism of purines. (D) Levels of the biogenic amines putrescine and spermidine. Bars represent SD. *p<0.05, **p<0.01, ***p<0.001. Abbreviations: TCA, taurocholic acid; TCDCA, taurochenodeoxycholic acid; TDCA, taurodeoxycholic acid; TMCA, tauro-β-muricholic acid.

Glycerophospholipids, major constituents of the cellular membrane,^27^ were not significantly affected by the HFD in either sex (choline, necessary for their synthesis, was not limiting; Figure 5A). There was a clear tendency for cholesterol esters to be decreased in the livers of male and female, young and aged mice fed an HFD, although the changes were only significant in the aged mice (Figure 5B). Furthermore, cholesterol esters are used for the synthesis of bile acids and followed a similar trend (ie, lower in HFD-fed mice, irrespective of sex and age; Figure 5B). Bile acids play a crucial role in the absorption of fat in the intestine, and their secretion from the gallbladder is enhanced by fat consumption.^28^

Other quantified metabolites included nucleotide catabolism molecules, biogenic amines, and amino acids. Previous studies have reported increased uric acid levels in patients with MASLD.^29,30^ The catabolism of purines generates uric acid from hypoxanthine and xanthine, which is catalyzed by xanthine dehydrogenase. No changes in *Xdh* gene expression were observed in HFD-fed male or female mice (Supplemental Table S1, http://links.lww.com/HC9/B22). Similarly, no changes in hypoxanthine and xanthine levels were observed in male mice (Figure 5C). However, both metabolites showed a trend to be elevated in aged female mice. This may represent a compensatory response to lipid overload and associated oxidative stress.^31^ Levels of the antioxidative molecule putrescine^32^ were increased upon HFD feeding in aged males (Figure 5D). Lastly, significant changes in nonessential amino acid levels (Ala, Gln, Cys, Gly, and Ser) and threonine were also observed between the groups, although no association was observed with diet, sex, or age (Supplemental Figure S4, http://links.lww.com/HC9/B21).

### Sex influences gene expression profiles in response to an HFD

**FIGURE 6:**
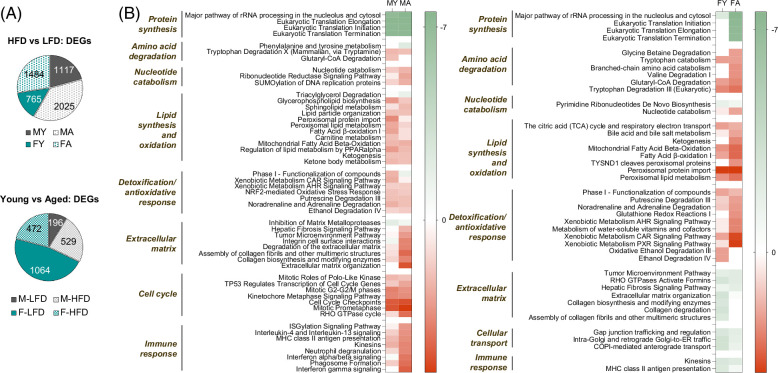
Gene ontology analysis. (A) Pie plot showing the total number of genes differentially expressed (DEGs) in mice fed an HFD relative to an LFD (top) and the number of DEGs in young mice relative to aged mice (bottom). (B) Canonical pathways of DEGs between mice fed an HFD relative to an LFD. The comparison analysis between DEGs in young and aged mice was carried out using the following filters: *p* value cutoff of 2.5 (log10) and *z* score cutoff of 1.5. The bar shows the activation *z* score. Abbreviations: DEG, differentially expressed gene; HFD, high-fat diet; LFD, low-fat diet.

The transcriptome was analyzed using RNA-seq in all groups. More than 12,000 genes were found to be expressed in the liver (Supplemental Table S1, http://links.lww.com/HC9/B22). Remarkably, in both sexes, there were more differentially expressed genes due to the HFD in aged mice (2025 males and 1484 females) than in young mice (1117 males and 765 females) (Figure 6A). In addition, males (young and aged) had significantly more differentially expressed genes than females in response to HFD feeding. Instead, aging had a much stronger effect on gene expression in females than in males (196 in males and 1064 in females) (Figure 6A; Supplemental Figures S5 and S6).

**FIGURE 7:**
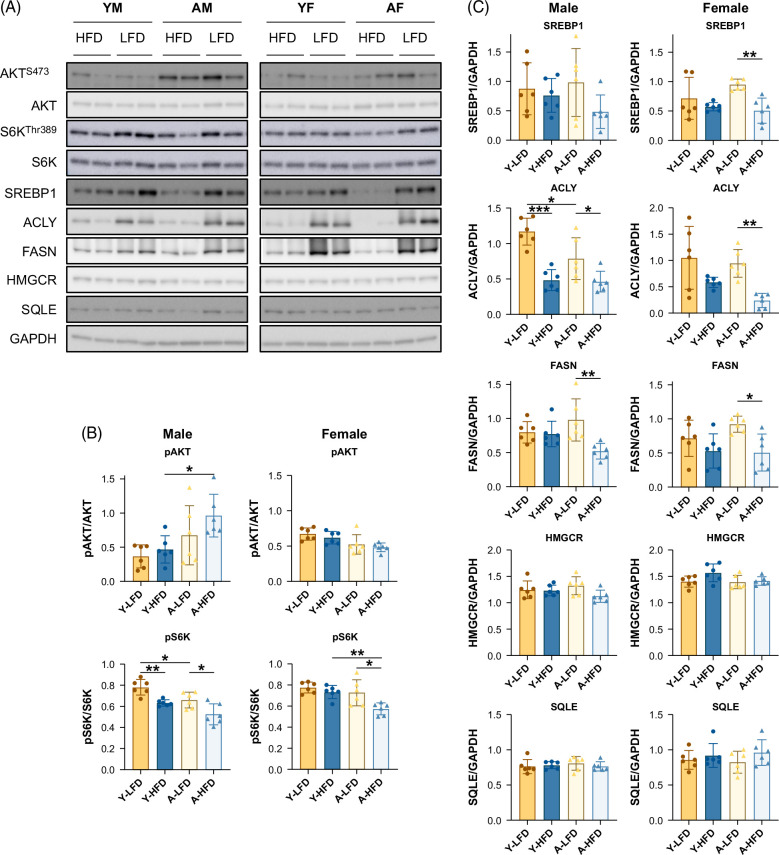
Validation of selected genes. (A) Western blot of molecules in the lipogenesis and cholesterol synthesis pathways. (B) Protein quantification of molecules in the lipogenesis pathway. (C) Protein quantification of molecules involved in cholesterol biosynthesis. Bars represent SD. *p <0.05, **p <0.01, ***p <0.001.

Gene clustering and biological theme enrichment were conducted using Ingenuity Pathway Analysis.^17^ In males (young and aged), the HFD induced a minor decrease in multiple small and large cytoplasmic ribosomal subunit genes, while in females, the differences were only significant in aged mice (Figure 6B; Supplemental Table S2, http://links.lww.com/HC9/B23). This decrease is likely to result from the combination of ER stress, which is associated with reduced protein biosynthesis^33^ and signaling through ribosomal protein S6 kinase (S6K), a major orchestrator of the response to nutrients that coordinates transcription and protein synthesis.^34^ S6K Th389 phosphorylation was moderately decreased in the liver of HFD-fed mice (Figure 7).

A large set of enriched categories included lipid metabolism (Figure 6B). Genes involved in mitochondrial fatty acid oxidation, such as *Acadm*, *Acsl1*, *Acaa2*, and *Acat1*, were significantly upregulated up to 1.6-fold in male and female mice (young and aged) fed an HFD. Furthermore, peroxisomal fatty acid oxidation was enhanced (*Acaa1a*, *Acox1*, *Acot2*, *Acot8*, etc.). Genes involved in ketone body production were also upregulated (*Acss3* and *Hmgcs2*). In contrast, genes implicated in de novo fatty acid synthesis (*Acaca*, *Fasn*, *Acly*, and *Scd1*) were downregulated in all groups fed an HFD (Supplemental Table S1, http://links.lww.com/HC9/B22; Figure 7). In males, this was observed despite the presence of hyperinsulinemia and increased Akt phosphorylation (Figure 7), a signal that activates lipogenesis through SREBP1 (sterol regulatory element-binding protein 1) expression.^35^ Thus, the HFD overrode the effects of Akt signaling. No changes were observed in the expression of cholesterol biosynthesis genes (Supplemental Table S1, http://links.lww.com/HC9/B22; Figure 7). None of the genes involved in ceramide synthesis, including *Cers2*, *4*, and *6*, were affected by the HFD in males and females; however, genes implicated in the degradation of gangliosides (*Hexa*, *Hexb*, *Neu2*, and *Gm2a*), glycosphingolipids (*Psap*), ceramide biosynthetic process (*Sptssa*), ceramide glucosyltransferase activity (*Ugcg*), and catabolism of galactosylceramide (*Galc*), were significantly different between the control diet and the HFD in males, reflecting the larger impact of the HFD on ceramide metabolism in males (Figure 6B; Supplemental Tables S1 and S2, http://links.lww.com/HC9/B22).

Multiple genes involved in “lipid particle organization” were significantly upregulated in young and aged males fed an HFD (*Cidea*, *Cidec*, *Fitm1*, and *Fitm2*). In females, only *Fitm1* was significantly upregulated. In addition, *Clstn3*, whose gene product is involved in regulating droplet size,^36^ was upregulated in young (~4.4-fold) and aged males (~1.5-fold) but only in aged females (~2.4-fold). Genes involved in triglyceride, lysophospholipid, and phospholipid synthesis (*Gpat*, *Pctp*, *Lpgat1*, and *Agpat3*) were moderately increased in males and females, young and aged, while genes implicated in their degradation were largely downregulated (*Pnpla3* and *Lpl*) (Supplemental Tables S1 and S2, http://links.lww.com/HC9/B22).

Genes involved in detoxification and antioxidative responses were similarly altered in young and aged males and females. However, a striking difference between the sexes was the expression of genes involved in extracellular matrix organization (Figure 6B). In young females fed an HFD, multiple genes involved in cell adhesion, including collagen synthesis (*Col1A1*, *Col3A1*, etc.) and metalloproteinases (*Mmp2*, *Mmp12*, *Trem2*, etc.), were downregulated by up to 20-fold. In contrast, many of these genes (*Col1A1*, *Col3A1*, *Mmp2*, *Mmp12*, *Trem2*, etc.) were increased in aged males fed an HFD, together with genes that are markers of fibrosis, such as endothelin (*Edn1*) and PDGF receptor beta (*Pdgfrb*), which were increased by ~2-to 4-fold. *Pdgfrb* is a staple feature of HSC activation.^37^ The expression of these genes did not increase in aged females. In addition, multiple clusters involving the regulation of the cell cycle and chromosome segregation were significantly enriched in males: *Cdc20*, *Cdca8*, *Kntc1*, and *Cdk1* were upregulated 2.7-to 5-fold (Figure 6B; Supplemental Tables S1 and S2, http://links.lww.com/HC9/B22).

Finally, genes involved in regulating the immune response clustered in multiple categories in males (toll-like receptors, chemokines, interferon-regulatory factors, and interferon-response genes [ISGs]) and were more significantly enriched in aged mice than in young male mice, but not found significantly represented in females, young or aged (Figure 6B; Supplemental Tables S1 and S2, http://links.lww.com/HC9/B22).

### Gene expression alterations associated with aging

**FIGURE 8:**
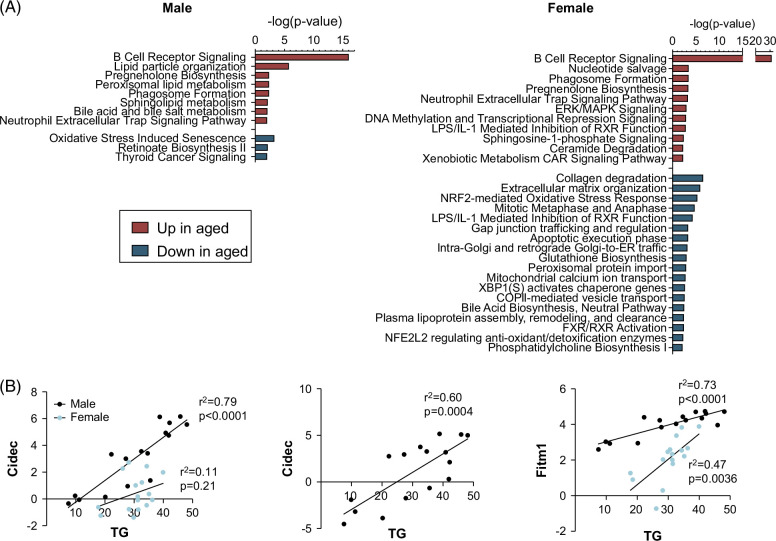
Gene expression changes in young versus aged mice. (A) IPA analysis of DEGs between young and aged mice fed an LFD. (B) Linear regression analysis showing a correlation between triglyceride levels (TGs, μM) and expression of *Cidea*, *Cidec*, and *Fitm1* (logCPM). All groups are included (LFD and HFD; young and aged; n = 4/group). Abbreviations: DEG, differentially expressed gene; HFD, high-fat diet; IPA, ingenuity pathway analysis; LFD, low-fat diet.

We then questioned whether changes in the transcriptome related to the aging process might promote a worse pathophysiology in aged mice when exposed to an HFD. In both sexes, the leading clusters included genes involved in immunoglobulin production (Figure 8A; Supplemental Table S3, http://links.lww.com/HC9/B24) (mostly *Igkv* and *Ighv* genes, encoding for kappa and heavy chains, respectively), probably reflecting the increase in size of foci of monotonous lymphoid cells (see the Histology section). Nevertheless, significant differences were apparent between the sexes. Lipid particle organization genes were upregulated multiple-fold in male mice. *Cidea* and *Cidec* expression was very low in young male mice fed the control diet but increased ~100-fold and 10-fold, respectively, in aged mice (Supplemental Table S1, http://links.lww.com/HC9/B22), correlating with liver triglyceride levels (Figure 8B). In females, *Cidea* was not detectable, and *Cidec* decreased rather than increased in aged mice. *Fitm1* expression increased ~3-fold in aged male and female livers, and its expression correlated with triglyceride levels in both sexes (Figure 8B).

In females, multiple genes involved in lipoprotein metabolism were significantly downregulated (Figure 8A; Supplemental Table S3, http://links.lww.com/HC9/B24). This cluster included genes such as *ApoC4*, *Ces3*, *Fgf21*, *Lpl*, *Pltp*, *Scarb1*, *Ubc*, and *Vldlr*. Liver-specific deficiency of the carboxylesterase *Ces3* leads to a reduction in serum triglyceride levels,^38^ and this gene was downregulated almost 5-fold in the livers of aged relative to young female mice. FGF21 is a hormone mainly produced by the liver that stimulates fatty acid oxidation, inhibits lipogenesis, and reduces the uptake of VLDL by decreasing VLDL receptor expression.^39^ *Fgf21* was reduced 4.6-fold in aged female livers. Furthermore, *Acot11*, an enzyme with thioesterase activity, significantly increased in aged females. Higher ACOT11 (THEM1) function in the liver has been linked to a reduced VLDL triglyceride secretion rate.^40^ Thus, it is possible that in aged females, changes in the expression of these genes contributed to reducing triglyceride loading onto VLDL and subsequent secretion into the circulation, in addition to influencing fatty acid oxidation. Furthermore, genes involved in regulating reactive oxygen species and cellular stress through mitochondrial Ca+-uptake (*Letm1*, *Micu1*, and *Micu2*) were downregulated in aged females.

Genes affecting cell adhesion were significantly downregulated in female mice, similar to the response to the HFD (Supplemental Table S2, http://links.lww.com/HC9/B23). Finally, a major difference between sexes involved clusters of genes with a function in cellular stress, which were significant only in females (Figure 8A; Supplemental Table S3, http://links.lww.com/HC9/B24). Multiple cytochrome (*Cyp*), glutathione (*Gsta1*, *Gstk1*, *Gss*, and *Gsr*), and chaperone (*Dnajb9*, *Lmna*, etc.) genes were expressed at lower levels in aged mice.

## DISCUSSION

Emerging clinical data are emphasizing the need for sex- and age-specific interventions to prevent the development of cardiovascular diseases in patients with MASLD.^10,11^ Understanding the etiology in males and females and the impact of aging is essential for the development of precision medicine treatments and a healthier aging process. In the present study, feeding mice an HFD resulted in obesity and glucose intolerance in young and aged males and females; however, males had a more severe phenotype, with the presence of hyperinsulinemia, and elevated levels of fasting blood glucose and ALT. In addition, the levels of diacylglycerol, C16:0 and C18:0 ceramides, and glycosylceramides, molecules associated with insulin resistance and fibrosis,^5,24,25,41^ were significantly increased in male mice but not in females. Furthermore, increased expression of genes involved in extracellular matrix organization, collagen synthesis, and the cell cycle was observed in males only. Proinflammatory cytokines play an important role in the pathophysiology of MASLD.^5,6^ Interferon response, toll-like receptor, and interleukin signaling pathways were upregulated in males but not in females. Thus, in response to an HFD, male mice developed a more severe metabolic disease than females and was exacerbated by age.

Females had higher baseline levels of triglycerides, sphingomyelin, and cholesterol esters, and the fatty acid oversupply from the HFD did not substantially increase particular lipid pools. However, aging affected the liver to a greater extent in females than in males. C16:0 and C18:0 ceramides increased in aged female mice but not in aged male mice. Acylcarnitines showed a clear trend to be lower in aged females, and aged females fed an HFD had significantly lower acylcarnitines than young females. These data suggest changes in fatty acid oxidation capacity and/or fatty acid transport into mitochondria, which may contribute to increased steatosis. Expression of genes implicated in fatty acid oxidation was not found to be significantly altered by age (including *Cpt1a*, *Cpt2*, *Crat*, and *Acsl*). Nevertheless, genes regulating lipoprotein metabolism (*Acot11*, *ApoC4*, *Vldlr*, *Ces3*, etc.), and *Fgf21*, which controls the fatty acid oxidation rate,^39^ were dysregulated. In addition, changes in oxidative stress gene expression were prominent in the aging female liver. Reduced expression of glutathione synthase (*Gss*, ~2-fold) and glutathione S-transferase alpha 1 (*Gsta1*; ~18-fold) suggests a lower capacity to protect the liver from reactive oxygen species. Furthermore, the trend toward increased purine catabolism (xanthine and hypoxanthine) was observed only in aged females. MASLD has been associated with increased levels of liver hypoxanthine and serum hyperuricemia, which are linked to oxidative stress.^29–31^ Overall, our data suggest changes in fatty acid oxidation/partitioning and oxidative stress, specifically in aging females. Estrogen plays an important role in regulating lipid metabolism in the liver. VLDL production and secretion, lipogenesis, and VLDL-TG clearance rates are regulated by estrogen.^42^ Although we did not analyze estrogen levels in this study, reduced concentrations of estradiol (E2) and progesterone in 12-month-old C57BL/6 mice have been reported.^21^ Our data suggest that the female mouse model used here is likely to have the hormonal changes characteristic of perimenopause, leading to metabolic derangements in lipid metabolism, as observed in humans.

Notable differences in gene expression and lipid distribution revealed underlying sex differences in liver physiology. Lipid droplets are fat-storing organelles mainly composed of a core of triglycerides and cholesterol esters, an external layer of phospholipids, and a coat of perilipins that decorate the surface. Cytoplasmic lipid droplets grow through the transfer of small lipid droplets to larger, more stable droplets, a process mediated by cell death-inducing DNA fragmentation factor-alpha-like effector (CIDE) family members A, B, and C.^43^ In a physiologically normal liver, *Cideb* is abundantly expressed, while *Cidea* and *Cidec* are barely detectable. However, in patients with MASLD as well as in mouse models of diet-induced obesity, increased expression of *Cidea* and *Cidec* has been observed.^44^ Furthermore, the level of *CIDEA* has been previously correlated with the degree of hepatic steatosis in humans and mice,^44–46^ and single nucleotide polymorphisms in this gene have been identified as risk factors for obesity.^47^ *Cidea* and *Cidec* expression was found to be increased in young and aged males fed an HFD, whereas only *Cidec* was upregulated in aged females. Furthermore, the expression of *Cidea* and *Cidec* was higher in aged male livers (~100-fold and 10-fold, respectively) but not in the female liver, suggesting that these genes play important roles in facilitating lipid storage in aging males.

Sex dimorphism in sphingolipid metabolism was also apparent. Ceramides are lipids present in the cellular membrane, forming ceramide-rich platforms that contribute to its fluidity and dynamics. These effects are dependent on acyl chain length, saturation, and the ratio of long-chain to very-long-chain acyl moieties.^48^ Long-chain and very long-chain ceramides have opposing effects on cell membrane fluidity.^49^ Long-chain ceramides (C16–C20) have been associated with insulin resistance, whereas very long-chain ceramides (C22–C24) have no effect.^24,25,50^ Remarkably, feeding an HFD resulted in a significant increase in C16:0 and C18:0 ceramides as well as in glycosylceramide levels, only in males. Glucosylceramide (HexCer), in particular, is associated with the transition from simple steatosis to steatohepatitis (metabolic dysfunction–associated steatohepatitis) and the development of fibrosis in patients.^41^

In conclusion, sexual dimorphism was observed in response to an HFD (Supplemental Figure S7, http://links.lww.com/HC9/B21). In males, HFD induces a more severe condition that is aggravated by aging. However, age-associated changes in liver physiology were most significant in females. Follow-up studies in females and older animals to identify the molecular mechanisms driving these age and sex differences are essential, as most research to identify the molecular mechanisms contributing to MASLD have used young male mice. With the increase in the aging of the global population, understanding the mechanisms driving the pathophysiology is vital for maintaining healthy aging in males and females.

## Supplementary Material
