# Supplementary material for: Sex- and age-associated factors drive the pathophysiology of MASLD
Source: Hepatol Commun. 2024 Aug 26;8(9):e0523. doi: 10.1097/HC9.0000000000000523 (PMC11357696; doi:10.1097/HC9.0000000000000523)
Supplement: Supplementary file 1 [file hc9-8-e0523-s001.pdf]

Sex and age-associated mechanisms drive the pathophysiology of MASLD

Ajay K. Yadav et al.

Supplementary Methods

Serum biochemistries. Blood glucose was measured with a AimStrip Plus glucometer (Germaine Laboratories, San Antonio, TX). All serum biochemistries were analyzed by the Center for Diabetes and Metabolic Diseases Translational Core. Insulin was analyzed using a mouse insulin ELISA kit (#10-1247-10, Mercodia, Uppsala, Sweden). Alanine aminotransferase (ALT), triglycerides, and cholesterol were measured using a Roche Integra 400 plus analyzer (#20764957322, #20767107322, #03039773190, respectively; Roche Diagnostics, Mannheim, Germany). Free fatty acids (FFA) were measured using a Randox Daytona Clinical Chemistry Analyzer (#FA115; Randox Laboratories, Crumlin, UK).

Tissue Histology. Liver tissue was fixed for 24 hours in 10% buffered formalin and transferred to 70% ethanol. Specimens were dehydrated through a graded series of ethanols (45 minutes per step), cleared in two changes of xylenes (45 minutes each) and infiltrated through four changes of melted paraffin (~60°C; 45 minutes each). The specimens were then embedded in melted paraffin and allowed to harden. Four-micron (4 μm) sections were cut using a rotary microtome equipped with disposable steel knives. Sections were flattened on a heated water bath, floated onto microscope slides and dried. Four-micron thick sections were cut from routinely processed paraffin embedded tissue and stained with hematoxylin and eosin or Masson's trichrome for histological examination. Sections of liver tissue snap-frozen in OCT compound were stained with Oil-Red-O. Processing and staining were performed by the Histology Services Core at Indiana University School of Medicine. The Non-alcoholic fatty liver disease Activity Score (NAS) was

assessed as described (sum of scores for steatosis, lobular inflammation, and hepatocyte ballooning) [1].

Western blotting. Liver tissue (~50 mg) was lysed in RIPA buffer (Thermo Scientific, Waltham, MA) containing protease and phosphatase inhibitors (Roche, Indianapolis, IN), as described previously [2]. Protein concentration was determined by BCA protein assay (Thermo Scientific). Proteins were separated in SDS-polyacrylamide Criterion gels (Bio-Rad, Hercules, CA) and transferred to 0.2-um polyvinylidene difluoride membranes (Bio-Rad). Primary antibodies were used in overnight incubations at 4°C (Supplementary Table 4), and secondary antibodies were added for 1 hour at room temperature. Blots were developed with ECL Western Blotting Substrate (Clarity^{TM}, Bio-Rad, or Pierce ECL, Thermo Scientific) and images were captured using a digital imaging system (BioRad). Bands were quantified by densitometry using ImageJ v1.48s, and results were normalized to control protein, as specified in the Figure legends.

References

[1] Kleiner DE, Brunt EM, Van Natta M, Behling C, Contos MJ, Cummings OW, et al. Design and validation of a histological scoring system for nonalcoholic fatty liver disease. Hepatology 2005;41:1313-1321.

[2] Jideonwo V, Hou Y, Ahn M, Surendran S, Morral N. Impact of silencing hepatic SREBP-1 on insulin signaling. PloS one 2018;13:e0196704.
