# Supplementary material for: Sex- and age-associated factors drive the pathophysiology of MASLD
Source: Hepatol Commun. 2024 Aug 26;8(9):e0523. doi: 10.1097/HC9.0000000000000523 (PMC11357696; doi:10.1097/HC9.0000000000000523)
Supplement: Supplementary file 2 [file hc9-8-e0523-s002.pdf]

Sex and age-associated mechanisms drive the pathophysiology of MASLD

Ajay K. Yadav et al.

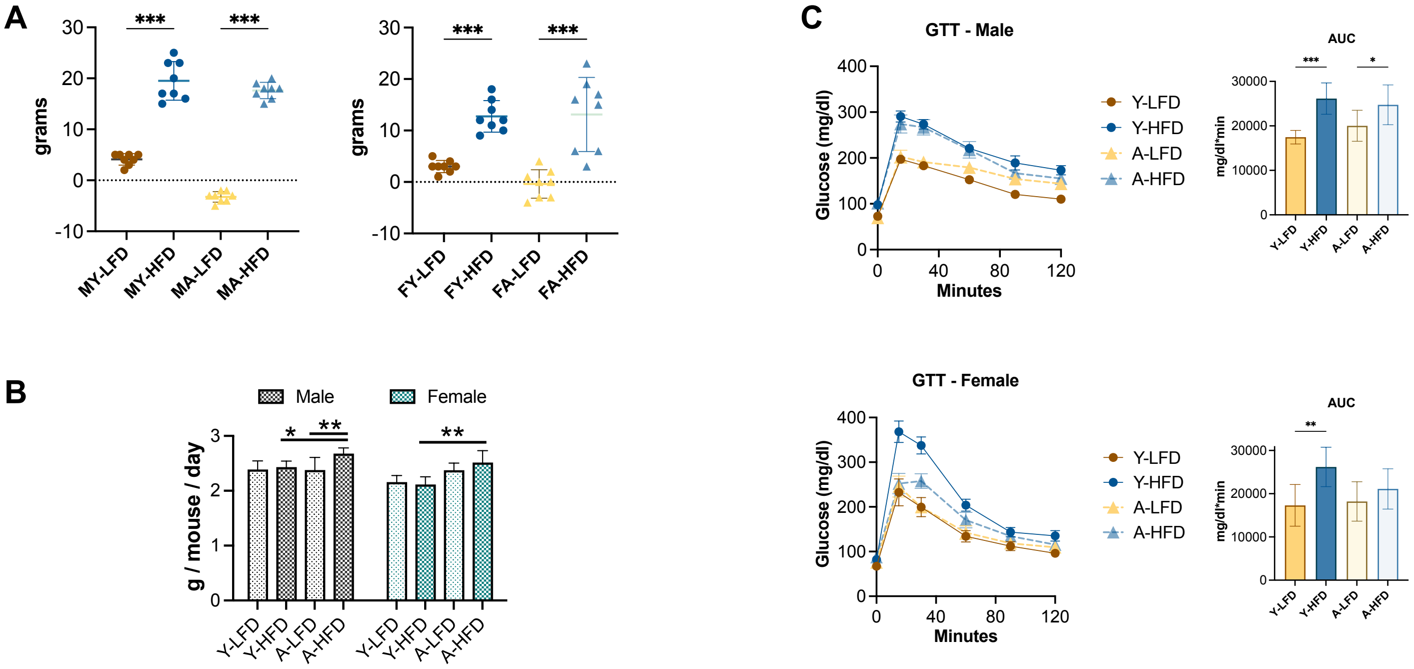

Supplementary Figure 1. Delta body weight change, food intake, and glucose tolerance test. **(A)** Delta body weight change (week 10-week 0). Bars represent standard deviation. **(B)** Food intake. Data represent the average of 8 measurements. Bars represent standard deviation. **(C)** Glucose tolerance test (n=7-8) and area under the curve of the GTT. Bars represent standard error of the mean. *p<0.05, **p<0.01, ***p<0.001.

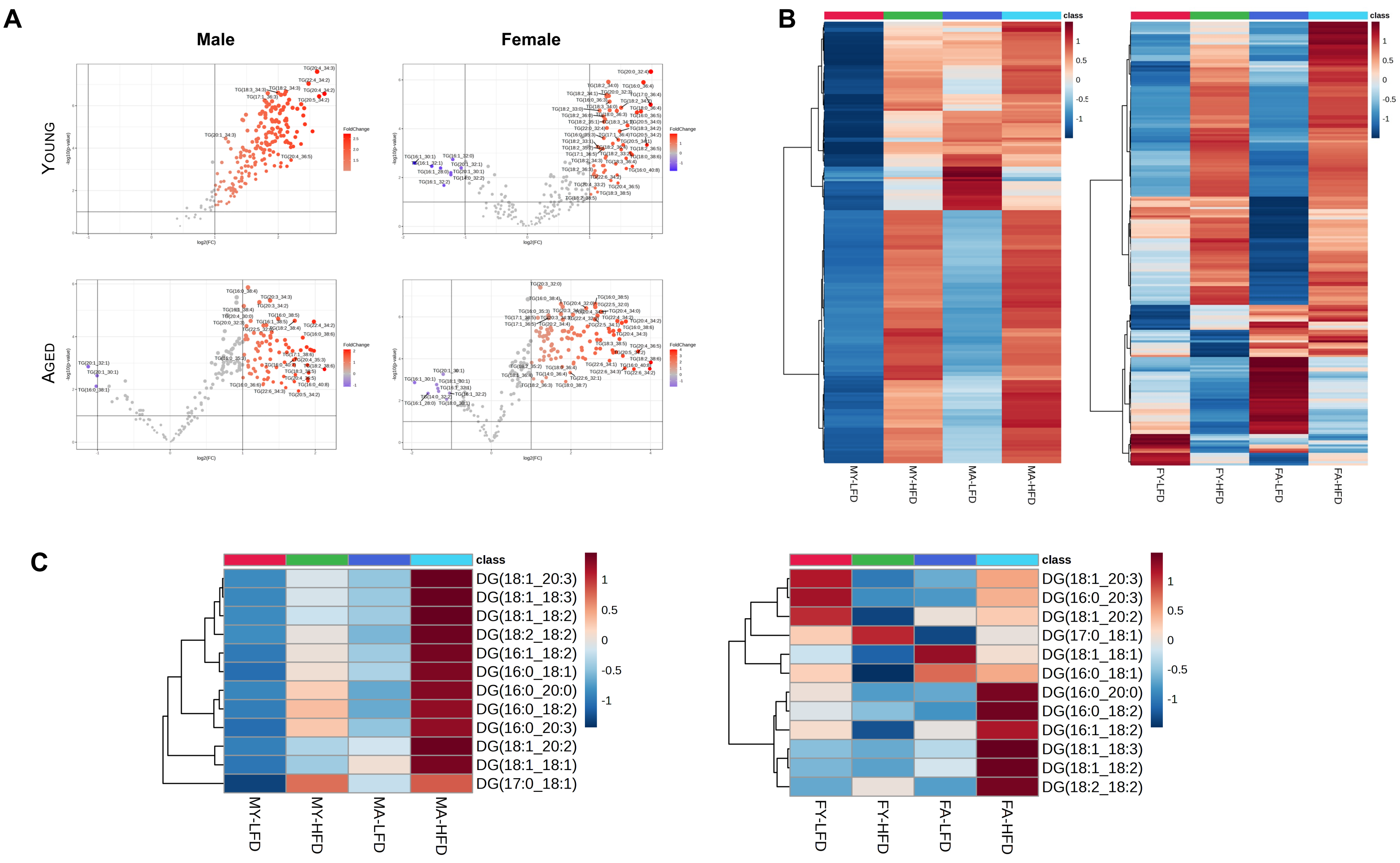

Supplementary Figure 2. Triglyceride classes in males and females fed a HFD or a LFD. **(A)** Volcano plot of triglyceride classes in mice fed the HFD relative to control mice fed the LFD. **(B)** Heatmap of triglyceride classes, showing more heterogeneity in the changes taking place upon feeding a HFD in females than males. **(C)** Heatmap of diacylglycerides; males respond to the HFD by increasing all classes, while females show shifts in abundance of specific chain lengths.

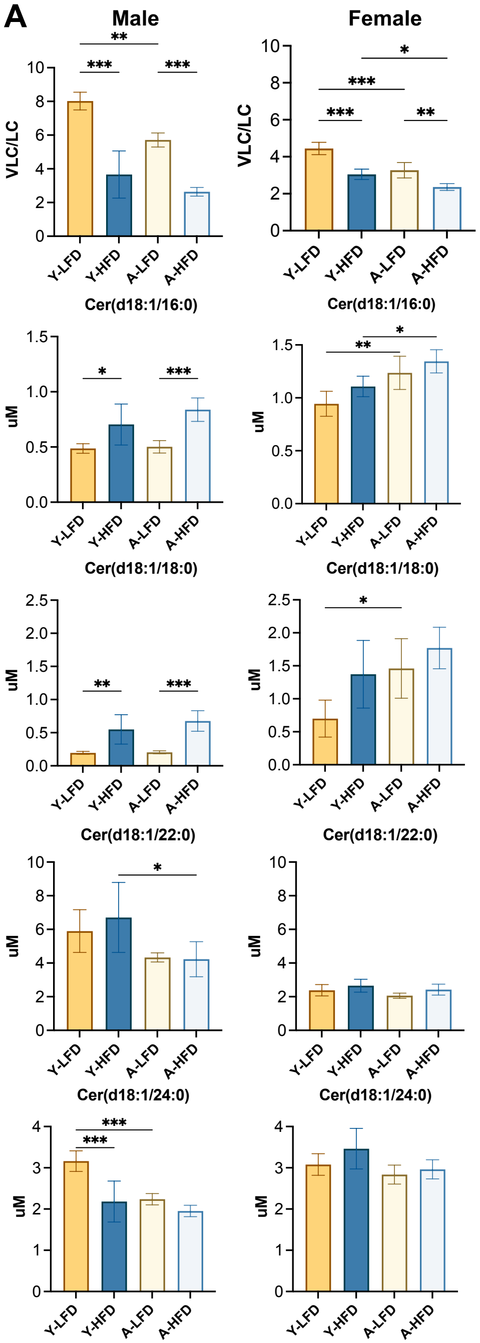

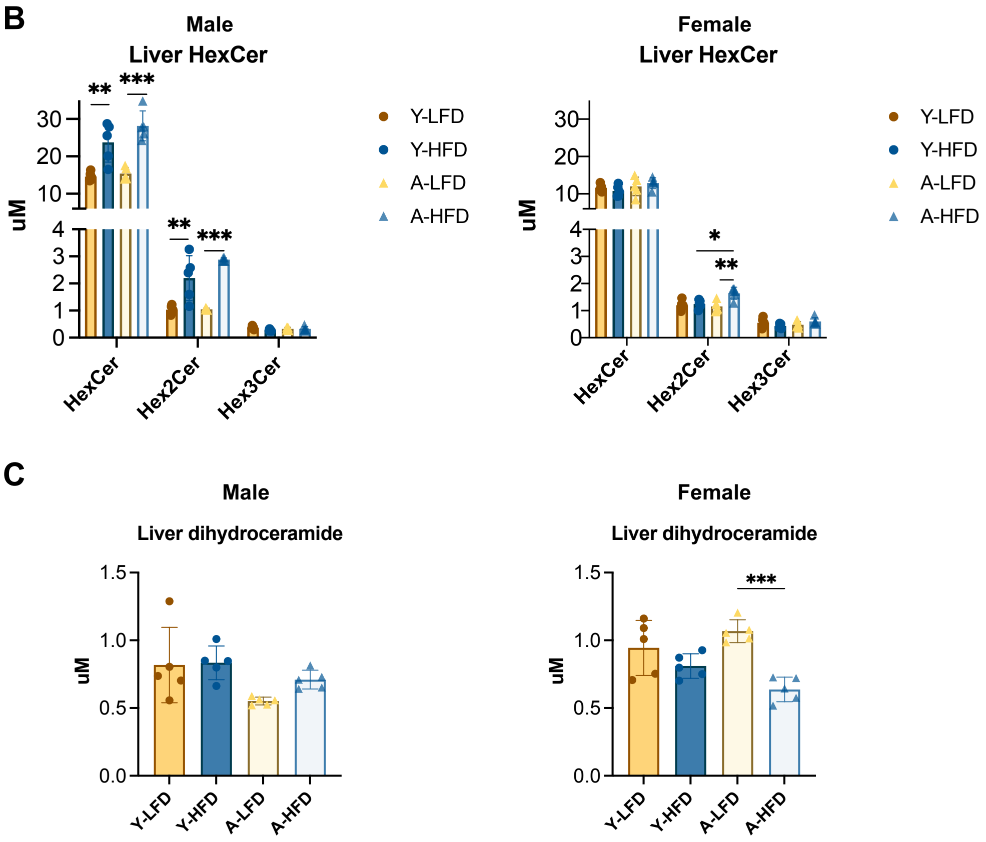

Supplementary Figure 3. Liver abundance of ceramide subclasses **(A)**, glycosylceramide subclasses **(B)** and dihydroceramides **(C)** in mice fed a HFD relative to control mice fed a LFD. Bars represent standard deviation. *p<0.05, **p<0.01, ***p<0.001. VLC, very long-chain, LC, long-chain. VLC and LC represent the sum of all VLC and LC subclasses identified in liver.

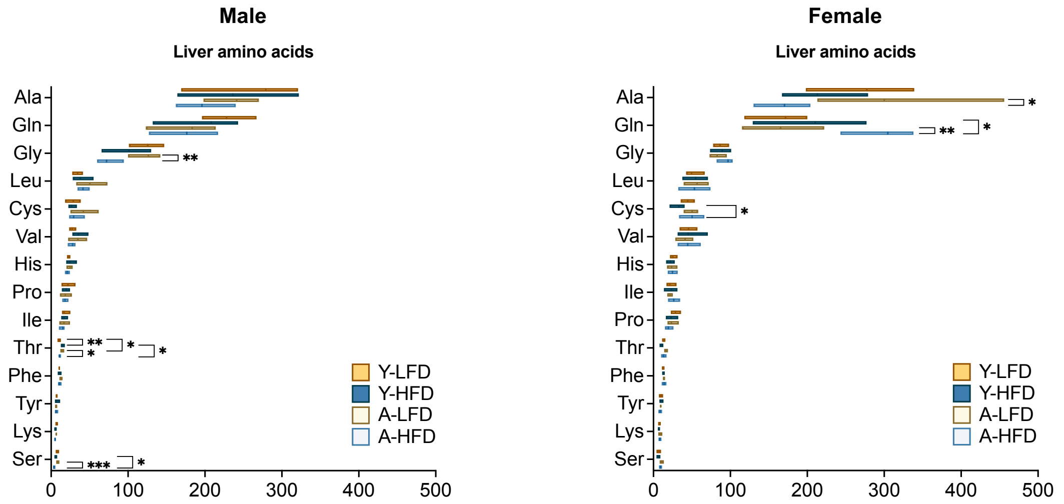

Supplementary Figure 4. Liver amino acid levels. Bars represent standard deviation. *p<0.05, **p<0.01, ***p<0.001.

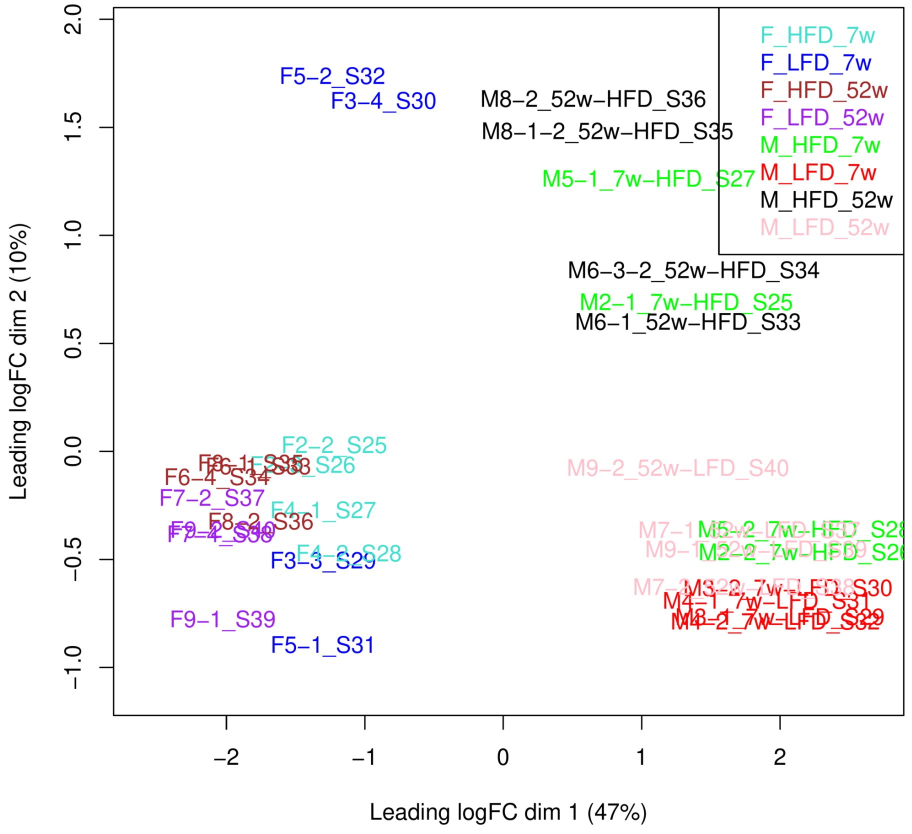

Supplementary Figure 5. MDS analysis of normalized RNA-seq read counts.

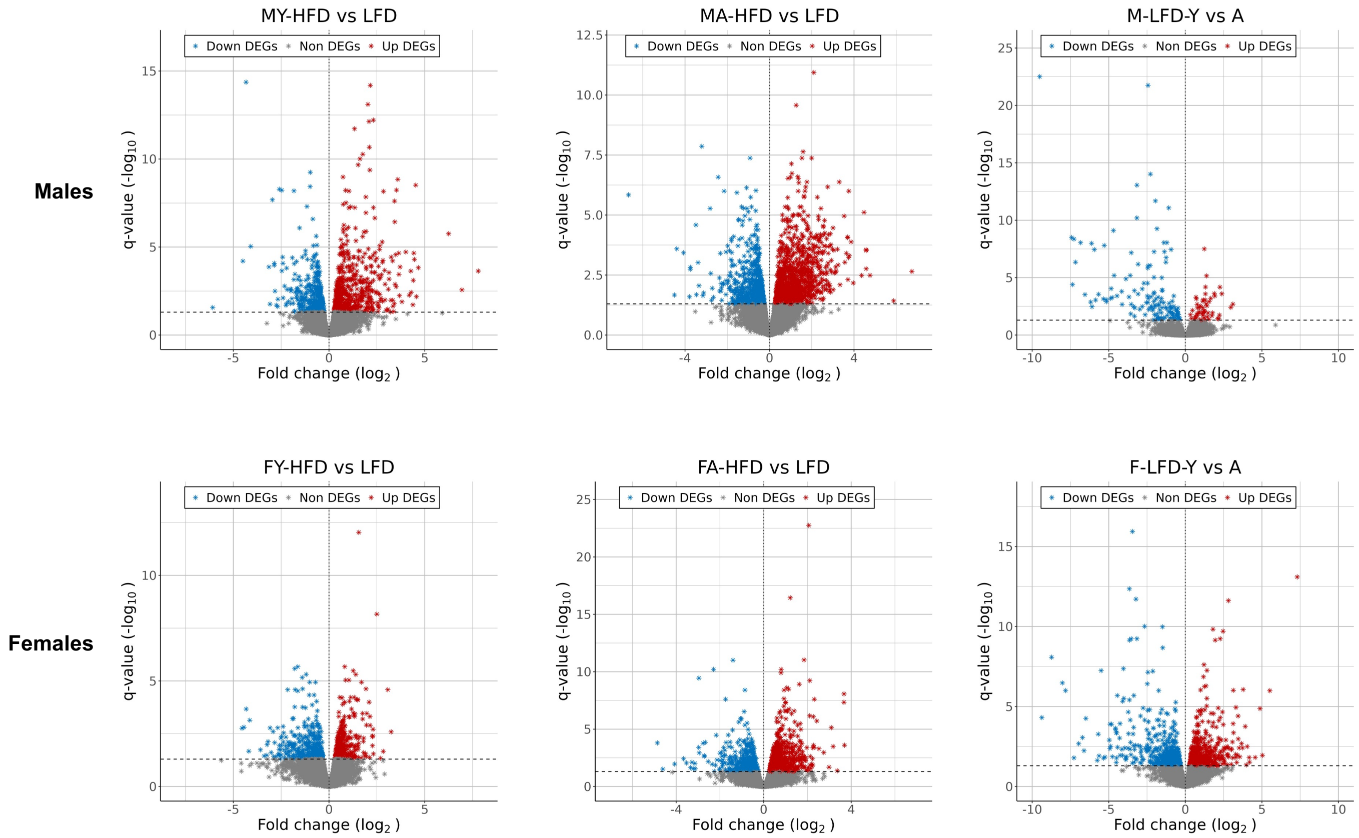

Supplementary Figure 6. Volcano plots of DEGs between mice fed a HFD and a LFD, and between young versus aged mice fed a LFD.

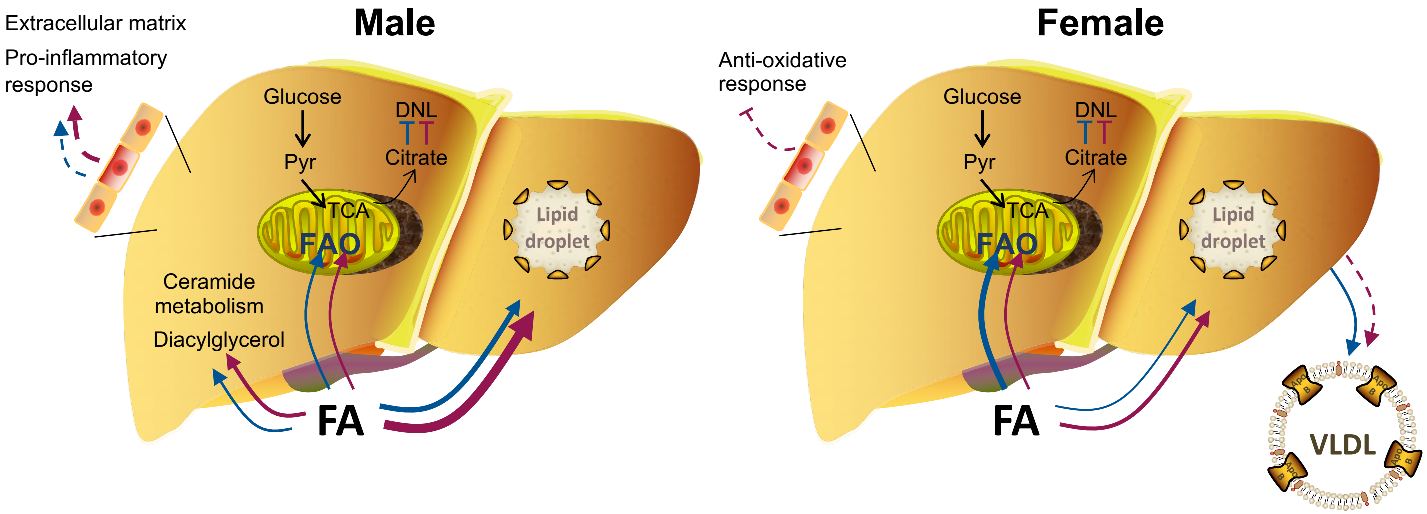

Supplementary Figure 7. Schematic overview of the differences between young and aged males and females.

- Young (blue arrows) and aged (purple arrows) males have a more severe metabolic disease than females upon feeding a HFD
- Females show minor changes in lipid pools in response to a HFD
- Aging affects females more profoundly, with decreased acylcarnitines, suggestive of changes in fatty acid oxidation (FAO) or transport into mitochondria, and altered anti-oxidative stress response and lipoprotein assembly gene expression (dashed arrows)
